# The Influence of ****γ****-Ray Irradiation on the Mechanical and Thermal Behaviors of nHA/PA66 Composite Scaffolds

**DOI:** 10.1155/2013/162384

**Published:** 2013-12-25

**Authors:** Fu You, Yubao Li, Yi Zuo, Jidong Li

**Affiliations:** Research Center for Nano-Biomaterials, Analytical & Testing Center, Sichuan University, Chengdu 610064, China

## Abstract

The aim of this study is to investigate the influence of sterilization process using **γ**-ray radiation on the melting behavior, crystallization behavior, thermal stability, and mechanical properties of nanohydroxyapatite/polyamide66 (nHA/PA66) scaffolds for bone tissue engineering. The results show that the melting temperature, degree of crystallization, thermal stability, and mechanical properties of the composite scaffolds increased with the enhancement of radiation doses from 25 kGy to 50 kGy, especially the irradiation dose of 50 kGy which imposed a remarkable effect on these properties. However, a reverse trend was found when the 100 kGy irradiation dose was applied. In general, a conclusion can be drawn that sterilization using **γ**-ray radiation with proper dose has no adverse effect on the properties of nHA/PA66 composite scaffolds.

## 1. Introduction

Porous scaffolds have been proved to play an important role in bone tissue engineering. The composite scaffolds have recently become a research hotspot due to its combination of the advantages of the different components in the composite. Based on the biomimic mechanism of the unique structure of hydroxyapatie/collagen in natural bone, a composite system consisting of nanohydroxyapatite (nHA) and polyamide 66 (PA66) was designed by our group [1] and had achieved favorable clinical effect [2]. Nanohydroxyapatite (HA) owns a considerable similarity in composition and structure to that of natural bone minerals, which consequently renders them osteoconductive and even osteoinductive, while the polymer phase of PA66 endows excellent mechanical properties to the nHA/PA66 composites. The nHA/PA66 composite scaffolds have been successfully developed by our group, which have exhibited desirable biocompatibility and osteogenesis [3].

It is well known that once the biomaterials have been manufactured, the devices must be sterilized prior to implantation. The sterilization methods used for biomedical devices include moist heat sterilization, radiation sterilization, and ethylene oxide sterilization. Moist heat sterilization is not suitable for the temperature-sensitive materials or for the products which need long-term storage. Ethylene oxide sterilization is one of the most commonly used methods which is known to cause little or no changes to the mechanical properties of the material [4]; however the potential toxicity resulting from ingredients residual limits its widespread application. Gamma radiation sterilization method, however, using typically a minimum of 25 kGy irradiation dose from a ^60^Co source, is widely used in the medical industry due to its efficiency and reliability, which can be carried out in air or in an inert environment.

The effect of ionization radiation on polymers is known to be one of the major sources for altering their internal structure, thus leading to a wide range of interrelated changes in their physicochemical properties. Such treatment may result in cross-linking and scission of the molecular chains of polymers or even degradation and destruction of the macromolecules, that is, formation of molecules with smaller chain lengths or change in the number or nature of the double bonds within the polymer backbone [5, 6]. While scission and crosslinking always occur to some degree in the materials subjected to gamma radiation, the presence of oxygen during radiation sterilization favored chain scission mechanisms and oxidative degradation. Thermal analysis is a vital analytical method in understanding the structure-property relationships and mastering the technology for molecular design and industrial production of different polymeric materials, especially inorganic reinforced polymer-based composites. Moreover, it is a useful technique to determine the thermal stability of the materials [7].

The sterilization is the last procedure before the application of biomaterials in clinic; it is important to well understand the influence of sterilization on the properties of the materials. The purpose of this study is to examine the influences of the *γ*-radiation sterilization treatment on the properties of nHA/PA66 porous scaffold, especially the mechanical and thermal properties. To study the effects of irradiation, both nonsterile and sterile nHA/PA66 scaffolds manufactured from the same batch were investigated.

## 2. Experimental

### 2.1. The nHA/PA66 Scaffolds Fabrication

All the chemical reagents used in this research were in analytical level. The nHA/PA66 composite slurry was prepared using the coprecipitation method in ethanol [8]. The nHA crystals slurry was prepared by our lab using wet synthesis method [9]. The nHA/PA66 composite slurry with 40 wt% (40 wt% nHA/PA66) and with 50 wt% (50 wt% nHA/PA66) nHA percentage was obtained by controlling the weight ratio (w/w) of nHA to PA66 during preparation.

The viscosity of the fabricated composite slurry was circumspectly adjusted to a relatively high value by evaporating solvent ethanol. Then the composite slurry was cast into a glass container with supporting metal bottom for better thermal conductivity and the phase change of ethanol from liquid to gas separation was thermally induced by heating the metal bottom to 90°C. Such a phase separation process produced the porous scaffolds. After complete solidification of the composite slurry, ultrasonic washing in deionized water, and being dried at 100°C for another 24 h, nHA/PA66 composite porous scaffolds were finally obtained.

### 2.2. Sample Preparation and Subsequent Irradiation

The prepared nHA/PA66 scaffolds with 40 wt% and 50 wt% nHA percentage were cut in different size and irradiation was conducted using a cobalt 60 energy source at room temperature. The doses applied in this study were of 25, 50, and 100 kGy.

### 2.3. Characterization

#### 2.3.1. Compressive Strength and Modulus of the Scaffolds

The compressive strength and modulus of the scaffolds were measured using a mechanical testing machine (AG-IC, SHIMADZU, Japan) following the method reported by Ma and Zhang [10] and Mathieu et al. [11]. Cubic specimens with side length of 10 mm were prepared. Five porous samples of each group were subjected to this test. The testing condition was at room temperature and 65% RH. The crosshead speed was set at 0.5 mm/min and the load was applied until the specimen was compressed to 70% of its original length. 

#### 2.3.2. Thermal Analysis

The thermal properties of nHA/PA66 scaffolds were tested by using a Netzsch STA 449 F3 simultaneous thermal analyzer under a nitrogen atmosphere with a sample mass of 4~5 mg. All samples were dried in vacuum oven at 80°C for 48 h to eliminate the humidity before testing. The thermogravimetric analysis (TG) curves of the scaffolds were recorded at a heating rate of 10°C/min and the temperature range was 20~550°C. The melting behaviour of all samples was assessed by differential scanning calorimetry (DSC) at a heating rate of 10°C/min from 20°C to 290°C. The melting temperature (*T*_*m*_) was noted as the maximum value of the melting peak. The crystallization behaviour of nHA/PA66 composites irradiated by different radiation doses was studied by using DSC. The melting samples were kept at 290°C for 5 min to eliminate heating history before cooling, and then brought down temperature to 150°C at a cooling rate of 10°C/min. Thermal stability was evaluated by derivative thermogravimetry (DTG) at a heating rate of 10°C/min. The scanning temperature scope was from 20°C to 500°C.

## 3. Results and Discussion

### 3.1. Microarchitecture

**Figure 1:**
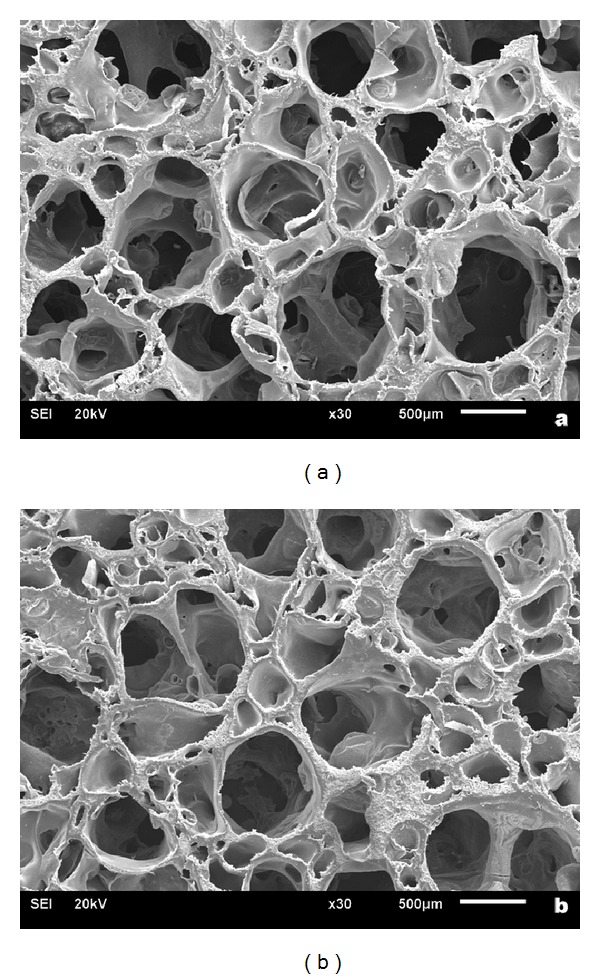
SEM photographs of the nHA/PA66 scaffolds with 40 wt% nHA (a) and 50 wt% nHA (b).

The microstructures of the fabricated nHA/PA66 composite scaffolds with 40 wt% nHA and 50 wt% nHA are similar to each other as shown in Figure 1. The range of pore size is from 300 *μ*m to 600 *μ*m, which might benefit cell ingrowth and bone tissue regeneration. 

### 3.2. Mechanical Properties

Table 1 shows the mechanical behavior of the irradiated and non-irradiated scaffolds. Both compressive strength and modulus of the nHA/PA66 scaffolds are enhanced with the increase in nHA content, which acts as the bioactive and reinforcing filler for the PA66 matrix. After irradiated with a dose of 25 kGy and 50 kGy *γ*-ray, the compressive strength and modulus of nHA/PA66 scaffolds with 40 wt% nHA and 50 wt% nHA content increased. Especially, a dose of 50 kGy *γ*-ray irradiation results in a more pronounced enhancement in compressive strength and modulus of the scaffolds in comparison to 25 kGy *γ*-ray irradiation. However, an obvious decrease in the compressive strength and modulus is found when a 100 kGy irradiation dose was applied. Additionally, the increase in mechanical properties of 40 wt% nHA/PA66 scaffolds is more pronounced than that of 50 wt% nHA/PA66 scaffolds. This can be attributed to the more polymer content in the composites, hereby resulting in more effects on structure and performance caused by *γ*-ray irradiation.

### 3.3. Thermogravimetric Analysis

**Figure 2:**
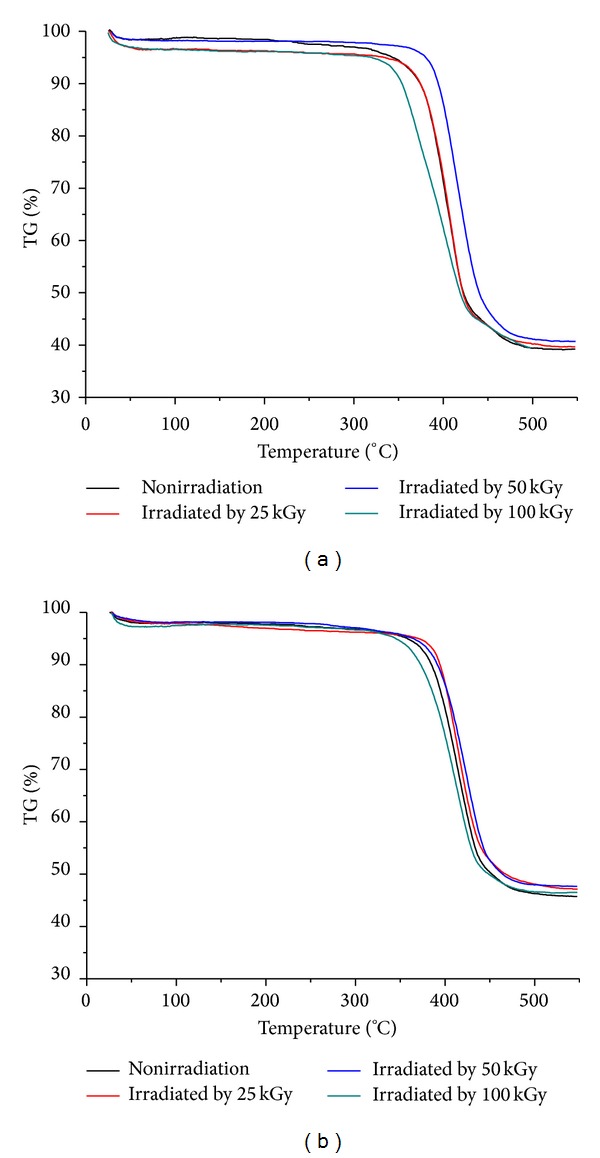
The effect of irradiation dose on TG curves for 40 wt% (a) and 50 wt% (b) nHA/PA66 composite scaffolds.

**Figure 3:**
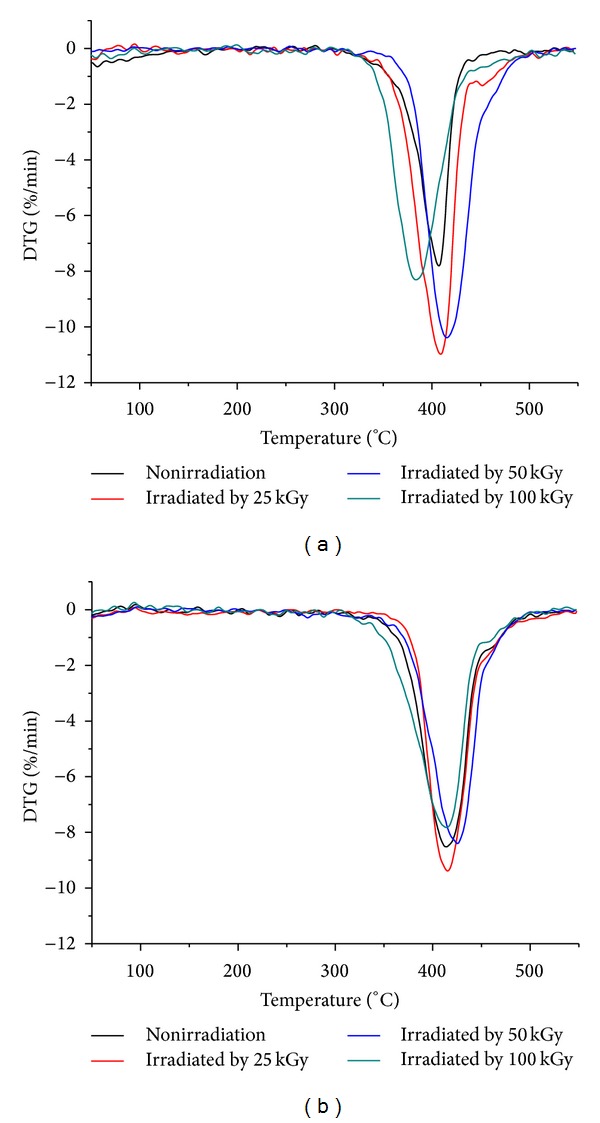
The effect of irradiation dose on DTG curves of 40 wt% (a) and 50 wt% (b) nHA/PA66 composite scaffolds.

Thermogravimetric curves of nHA/PA66 composites containing 40 wt% and 50 wt% nHA irradiated by different radiation doses are shown in Figure 2. The thermal degradation of the samples in most cases is a single-step process.  Figure 2(a) shows the effect of radiation doses on the thermal stability of 40 wt% nHA/PA66. The thermal stability of the composites is improved with the increase in radiation doses from 0 kGy to 50 kGy, whereas a radiation dose of 100 kGy leads to an obvious decrease in thermal stability as evidenced by a completely reverse trend of the rage of degradation temperature. The same trend is also observed in DTG curves (Figure 3(a) and Table 2); the peak of the scaffolds irradiated by 50 kGy shifts to higher temperature compared to those of other groups, suggesting the higher thermal stability of the composite scaffolds irradiated by 50 kGy than that of the other groups. Similarly, it is also found from Figure 2(b), Figure 3(b), and Table 3 that the irradiation dose has a similar effect on 50 wt% nHA/PA66.

### 3.4. DSC Analysis

To evaluate how the thermal properties of the materials are affected by sterilization with high-energy radiation, DSC was used to determine the *T*_*m*_ and the amount of crystallinity of both non-irradiated and irradiated porous scaffolds.

#### 3.4.1. Melting Behaviour

**Figure 4:**
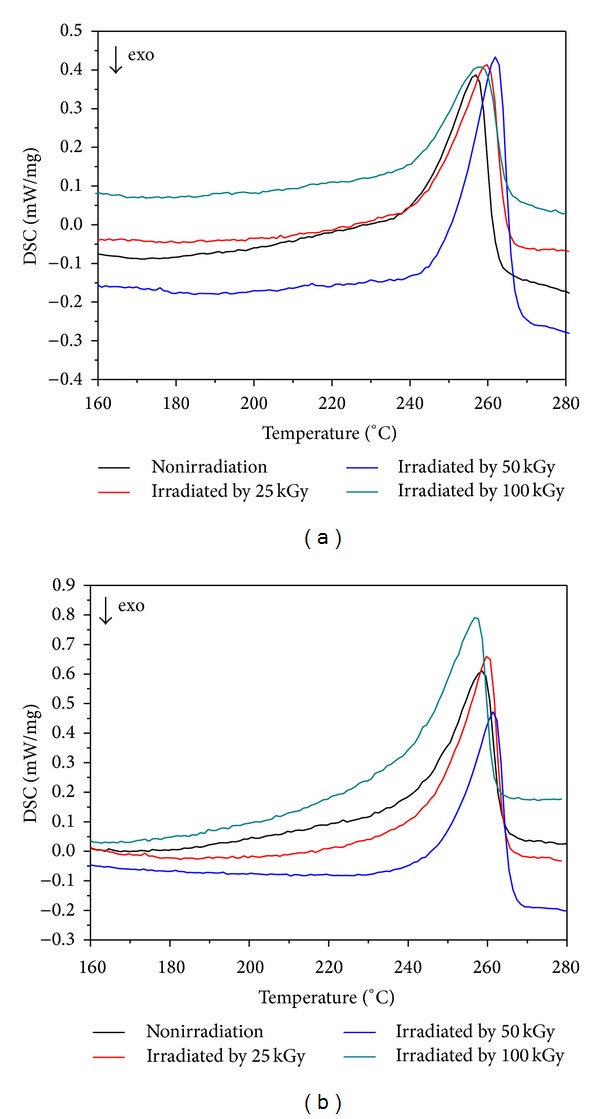
The effect of irradiation dose on curves for the melting of 40 wt% (a) and 50 wt% (b) nHA/PA66 composites.

Figure 4 shows the effect of irradiation dose on the melting curves of 40 wt% and 50 wt% nHA/PA66 composite scaffolds. All nHA/PA66 samples show only one melting peak. The melting temperature, *T*_*m*_, noted as the maximum value of the melting peak, is shown in Table 4.

For the 40 wt% nHA/PA66 group, it can be seen that the PA66 component experiences an increase in the melting temperatures after 25 kGy and 50 kGy irradiation, followed by an obvious decrease that resulted from irradiation dose of 100 kGy. The same trend is also observed for the 50 wt% nHA/PA66 group.

#### 3.4.2. Crystallization Behaviour

**Figure 5:**
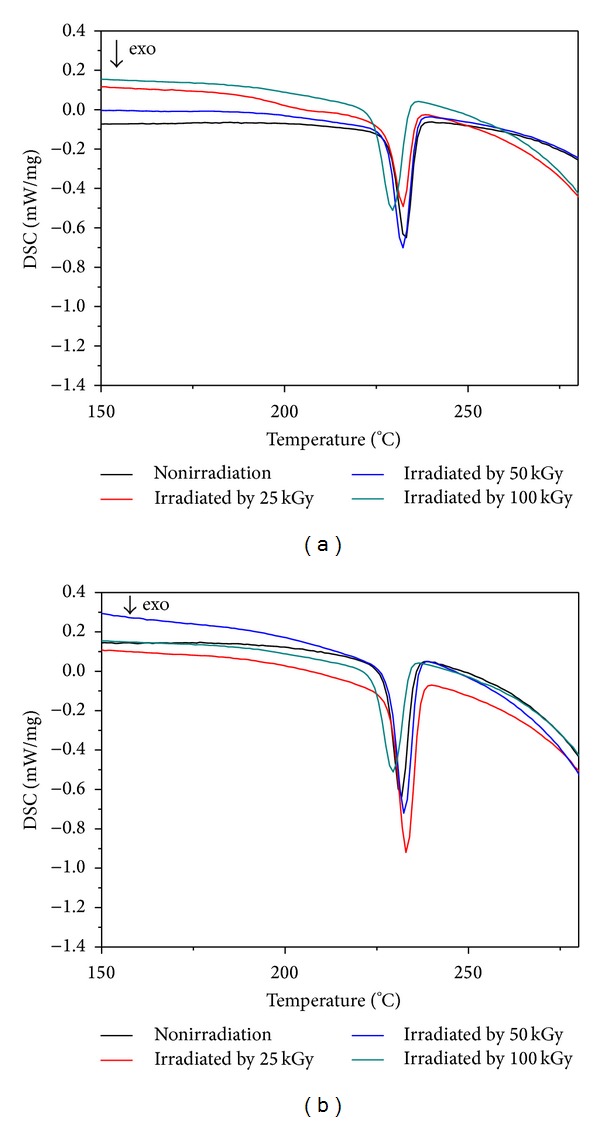
The curves for the crystallization behaviour of 40 wt% (a) and 50 wt% (b) nHA/PA66 scaffolds irradiated by different doses.

Figure 5 shows the DSC curves for the crystallization behaviour of nHA/PA66 samples. The values of the temperature for initial crystallization (*T*_ci_), temperature for maximum crystallization (*T*_*c*_, max), heat for crystallization (Δ*H*_*c*_), and the degree of crystallization (*X*_*c*_) nHA/PA66 composite scaffolds are listed in Table 5.

The degree of crystallization of the PA66 component was determined by using the following equation:

(1) $$\begin{array}{c} X_{c}\%=\frac{\Delta H_{c}\times 100}{\Delta H_{c}^{0}}, \end{array}$$

where *X*_*c*_ is the degree of crystallization of PA66 component, Δ*H*_*c*_^0^ is the heat of crystallization for 100% crystalline PA66, which is 188 J/g [12], and Δ*H*_*c*_ is the heat of crystallization for nHA/PA66 composite scaffolds.

The changes in heat for crystallization and degree of crystallization were modest for all samples over the entire radiation dose interval, as shown in Table 5. When the scaffolds are irradiated, chain shortening (chain scission) and cross-linking reactions take place simultaneously, but to different extents, which most likely occur more often in the amorphous phase. After samples are irradiated under the dose of 25 kGy and 50 kGy, the heat for crystallization (Δ*H*_*c*_) and the degree of crystallization (*X*_*c*_) show a slight increase.

The change of thermal behavior and the mechanical properties of the nHA/PA66 composite scaffolds after irradiation may be explained as follows. In the process of irradiating, two main reactions may occur in the composite, especially in the polymer phase, that is, cross-linking and chain shortening (chain scission) reactions, both of which take place at the same time. The chain shortening process results in break-down of macromolecular chains but thereby leads to a high melting temperature most likely owing to the more tightly packing and rearrangement of shortened polymer chains. However, when samples were irradiated by the radiation dose of 100 kGy, the melting temperature shows a sharp decrease for both 40 wt% nHA/PA66 and 50 wt% nHA/PA66 groups. This can be explained by the high-energy irradiation in air lead to progressive reduction in molecular weight and eventual degradation to nonpolymeric materials to some extent [13–15].

The variation of Δ*H*_*c*_ and *X*_*c*_ as a function of irradiation dosage can be also explained by the above-mentioned chain scission mechanism. When the samples were irradiated by intermediate doses in air, the polymer chains are broken down, thus resulting into shorter molecular chains that are able to rearrange in a more ordered manner and further leading to a higher crystallinity of PA66 phase within the composites. Meanwhile, the irradiation enhances the cross-linking degree of the polymer chains, which results in the increase in the crystallinity. When nHA/PA66 scaffolds were irradiated by the irradiation dose of high value, for example, 100 kGy, the oxidative degradation may occur, which may produce more shortened chains, enhance the chain mobility, and consequently lower the heat for crystallization (Δ*H*_*c*_) and the degree of crystallization (*X*_*c*_).

The enhancement of mechanical properties of the scaffolds after 25 kGy and 50 kGy irradiation can be explained by the cross-linking and chain scission mechanisms increasing the crystallinity of the polymer in the composite. Also, the presence of hindering groups like nHA attaches to the main chains of polymer through hydrogen-bonding in favor of chain scission [16, 17], which may augment the effect resulting in the increase of mechanical properties after an intermediate dose of irradiation. Irradiating polymers with gamma radiation of high value of 100 kGy lead to a decrease in compressive strength and modulus may be caused by oxidative degradation which usually coexists with chain scission in the presence of oxygen resulting in the decrease in the crystallinity. When being irradiated by 100 kGy, the effect of oxidative degradation may be augmented [18]; thus the mechanical behavior exhibits an obvious decrease.

## 4. Conclusions

Sterilization by *γ*-ray radiation has been widely used in the medical industry due to its efficiency and reliability. The current study investigated the influence of *γ*-ray radiation on the thermal and mechanical properties of nHA/PA66 composite scaffolds for bone tissue engineering. TG, DSC, and mechanical testing were employed to assess the influence. The results show that an intermediate dose of irradiation could enhance the mechanical property and thermal stability of composites, while a high dose leads to a decrease in compression strength and thermal property of the scaffolds. The change of thermobehavior and the mechanical properties can be explained by the occurrence of cross-linking and chain scission reactions resulting from the irradiation procedure.

## Figures and Tables

**Table 1:** Compressive strength and modulus of irradiated and nonirradiated scaffolds.

Samples	Irradiation dose (kGy)	Compressive strength (MPa)	Compressive modulus (MPa)
40 wt% nHA/PA66	0	1.43 ± 0.45	17.12 ± 3.54
	25	1.57 ± 0.51	15.61 ± 5.57
	50	2.27 ± 0.19	21.82 ± 4.12
	100	1.01 ± 0.17	4.99 ± 10.57

50 wt% nHA/PA66	0	2.04 ± 0.22	20.58 ± 2.12
	25	2.27 ± 0.38	21.58 ± 5.86
	50	2.31 ± 1.07	37.63 ± 2.70
	100	1.29 ± 0.54	32.26 ± 4.16

**Table 2:** Thermal properties of 40 wt% nHA/PA66 composites from TG and DTG curves.

Irradiation dose (kGy)	*T*_*i*_ (°C)	*T*_*f*_ (°C)	Δ*T* = *T*_*f*_ − *T*_*i*_ (°C)	*T*_max⁡_ (°C)
0	345	486	141	410
25	345	484	139	411
50	360	486	126	420
100	325	461	136	379

**Table 3:** Thermal properties of 50 wt% nHA/PA66 composites from TG and DTG curves.

Irradiation dose (kGy)	*T*_*i*_ (°C)	*T*_*f*_ (°C)	Δ*T* = *T*_*f*_ − *T*_*i*_ (°C)	*T*_max⁡_ (°C)
0	350	468	118	415
25	350	471	80	417
50	391	473	81	424
100	375	465	90	415

**Table 4:** The melting temperature of 40 wt% nHA/PA66 and 50 wt% nHA/PA66 irradiated by different doses.

Samples	40 wt% nHA/PA66				50 wt% nHA/PA66			
	0 kGy	25 kGy	50 kGy	100 kGy	0 kGy	25 kGy	50 kGy	100 kGy
*T*_*m*_ (°C)	255	259	263	257	259	261	263	255

**Table 5:** Thermodynamic parameters of 40 wt% nHA/PA66 and 50 wt% nHA/PA66 irradiated by different doses.

Samples	40 wt% nHA/PA66				50 wt% nHA/PA66			
	0 kGy	25 kGy	50 kGy	100 kGy	0 kGy	25 kGy	50 kGy	100 kGy
*T*_ci_ (°C)	255	259	263	257	259	261	263	255
*T*_*c*, max_ (°C )	235	231	229	227	226	230	228	223
Δ*H*_*c*_ (J)	44	43	51	36	37	41	49	31
*X*_*c*_ (%)	23	23	27	19	19	22	26	16
